# The significance of resilience in mental health promotion of marriage immigrant women: a qualitative study of factors and processes

**DOI:** 10.1186/s12905-020-00945-3

**Published:** 2020-04-28

**Authors:** Yeonjae Jo

**Affiliations:** Dong-A University, College of Nursing, G05-510, Daeshingongwon-ro 32, Seogu, Busan, 49201 South Korea

**Keywords:** Marriage immigrant women, women’s health, Mental health, Access to service, Resilience theory, Qualitative research

## Abstract

**Background:**

This study explores a series of processes in which marriage immigrant women achieve positive mental health status after experiencing various marriage- and migrant-related difficulties through the framework of resilience theory. As marriage immigrant women face greater barriers to public health services than non-immigrant women, it is necessary to understand the related factors, process, and context to address these barriers and strengthen available assets.

**Methods:**

A qualitative case study design was used with the phenomenological approach. Eleven mental health promotion program managers and 12 marriage immigrant women from who experienced resilience were recruited from four public-funded multicultural community centers in Seoul and Gyeonggi-do, South Korea, between December 2015 and March 2016. Using data from in-depth semi-structured face-to-face interviews, the author applied theme analysis informed by the resilience theory in order to identify factors that affect resilience and its development process.

**Results:**

Findings indicated that the process of resilience follows enduring difficulties, collapse of stability, access to professional help, professional and social support, and experience of growth. A combination of the staged process of growth, absence of partner support, children as a driving force for change, the need for economic activity, factors affecting difference in growth: satisfaction levels of women’s need for recognition, respect, and reward, and level of spousal support were identified as factors affecting marriage immigrant women’s resilience.

**Conclusions:**

Spouses, children, and economic activity play key roles in resilience in positive and negative ways. The existing information barrier should be addressed at a structural level to improve the mental health of marriage immigrant women, and the optimum time for intervention is suggested within 2 years post-migration. Efforts to build supportive relationships with Korean spouses and meet the women’s needs for recognition, respect, and reward may also help promote these women’s resilience.

## Background

As a result of the Asian economic crisis, female marriage migration has increased from Southeast Asia to South Korea. There has been an increase in marriages of immigrant women over the last few decades; in 2005, the number of marriages between South Korean men and foreign women accounted for 9.8% of the total marriages in the country (314,304) [1]. The cumulative number of marriage immigrant women reached 274,282 in 2018 [1].

Research on marriage immigrant women tends to focus on their vulnerabilities to stressors, mental distress, physical abuse, and social isolation [2–5]. Most of these women immigrate to a foreign country in their early 20s and 30s; thus, they face challenges associated with immigrant adjustment, including adjusting to a new marriage, pregnancy, childbirth, and employment [6, 7]. Marriage immigrant women need social support for settling in South Korean society [8] and have a direct and indirect influence on family and social health. Often marriage immigrants find themselves isolated in their new country without the support networks they are used to having in their country of origin or proficiency in the Korean language. The inability to speak or read the language of the new country has a significant negative effect on the social integration of female migrants [9]. This places married women in a position of dependence on their South Korean spouses, reducing their autonomy. There are a number of studies on the mental health problems these women face due to changes in the living environment, lifestyle, and cultural adaptation stress, such as depression and anxiety [10, 11]. Studies have repeatedly expressed that female migrants tend to experience more cultural adaptation stress and depression [12–14]. Gender differences in the psychological adaptation of migrants tend to be more pronounced when the differences in gender role expectations between the two cultures are greater, especially in the more stratified societies. Women find greater difficulty adapting and are more likely to have psychological symptoms such as depression, as compared to men [15–17].

In response to the increasing number and the integration needs of marriage immigrants, the Korean Government has adopted social welfare policies to facilitate their adjustment based on the rationale that they have provided a segment of Korean men with the opportunity to continue their family line [18]. For example, the Korean National Health Insurance Program covers marriage immigrants who hold a valid spouse visa, providing financial assistance or waiving the health insurance premiums when they give birth [19]. The Ministry of Health and Welfare and Ministry of Gender Equality and Family sponsor 218 public multicultural community centers that provide Korean language education programs, psychological counseling, and art therapies to support immigrants’ adjustment in Korea. However, due to lack of funds and limited capacity, these centers have less accessibility, and the number of counseling sessions is limited to 10 or fewer. Furthermore, they focus primarily on education rather than intervening in individual cases [20]. Building healthy public policy, creating supportive environments, strengthening community actions, developing personal skills, reorienting health services, and caring holism and ecology were identified as priority action areas of health promotion in the First International Conference on Health Promotion in Ottawa, Canada [21]. The mental health of marriage immigrant women should be viewed in relation to the internal resources and external support for the individual, given the holistic and ecological perspective that has been emphasized in the Ottawa Charter [21]. In addition, it is necessary to study not only the negative but also positive factors influencing mental health and the process of recovery and growth from mental health problems [22].

Lately, as more studies have been conducted with people recovering or growing from negative life events or difficulties [23–26], there is increasing interest in resilience theory to explain these processes and results. The concept of resilience can be defined as the process and consequences of working protective factors that enable positive adaptation, recovery, and growth from difficulties [27].

Several researchers have used quantitative research methods to identify protective factors regarding resilience [28–30]; however, the development process of resilience and its context are not identified in these studies. Additionally, existing studies of immigrant women show a lack of application of resilience theory. Unlike traditional medical models that focus on weaknesses, deficiencies, and pathogenic factors, resilience theory focuses on possibility, assets, strength, and supportive aspects of individuals’ environment that can empower them to gain control over their life effectively [29]. Furthermore, resilience theory considers a person an active rather than a passive subject [20]. To understand the difficulties experienced in the specific context of immigrant women’s marriage and how immigrants’ inner resources and external protective factors promote mental health throughout the resilience process, in-depth, qualitative research is required.

The resilience of marriage immigrant women has received little attention in political and research agendas. This study adds to the existing literature with a new approach focused on the analysis of resilience protective factors, their context, and the resilience process of marriage immigrant women. As such, qualitative research was used to examine the experiences of marriage immigrant women, analyzing several categories of factors that may promote or harm resilience, driving forces of change, and key influencers on growth and ability to thrive.

In this context, the purpose of this study is to examine the process of resilience in the mental health of marriage immigrant women and its influencing factors. The results may then provide a basis for complementing and improving existing mental health promotion policies, services, and new public health strategies.

## Methods

### Participants

Eleven mental health promotion program managers with different professional specialties were selected from four public-funded multicultural community centers in Seoul and Gyeonggi-do, South Korea. These programs were sponsored by The Ministry of Health and Welfare and Ministry of Gender Equality and Family that aimed to support for early adaptation and stable settlement of marriage immigrant women and their families. Twelve marriage immigrant women who had indicated to program manages that they were happy to participate and who were able to be contacted by the researcher were invited to participate in the face-to-face interview, and all were interviewed.

### Design and data collection techniques

This is a qualitative case study focusing on a phenomenological approach. By allowing immigrant women the opportunity to describe their experiences and the meanings they attribute to them, the researcher can capture the diversity and complexity of their perceptions of the resilience process and the related factors. As Lahtinen et al. (1999) suggest, key concepts needed in the planning, evaluation, and monitoring of mental health promotion and prevention programs/policies were considered to evaluate the contents and quality of the current mental health promotion programs for marriage immigrant women. Critical case selection was made considering comprehensive factors such as individual, family, social, and cultural factors to ensure the quality of mental health promotion programs for marriage immigrant women [31].

Data were collected from two groups, women and program managers. Two programs were decisively chosen, and additional cases were secured through a snowballing method using the institutions as a focal point. The selection criteria of the program manager included having worked with migrants for more than 1 year, currently working, voluntary participation, and work as a mental health promotion program manager. The selection criteria of marriage immigrant women included having experienced participating in a mental health promotion program for more than 1 year, recommended by program manager as recovered or resilient, voluntary participation, and willing to talk/share about their experience. Program managers and marriage immigrant women who did not meet the selection criteria were excluded. Each program manager was selected first and then recommended immigrant women for participation. Immigrant women meeting the selection criteria were contacted by their program manager, informed about the study, and asked to consider participating. Immigrant informants willing to volunteer were identified and recruited through program managers first. Then the researcher contacted them via telephone, re-informed them about the study and their rights, and confirmed their voluntary participation. A suitable time and place for the interview was then arranged. The final sample size was determined by data saturation, which was verified when no new data relevant to the study were found.

In-depth, semi-structured face-to-face interviews were conducted by the author, a native Korean speaker, between December 2015 and March 2016 using standardized protocol. The research questions were as follows: How can marriage immigrant women attain resilience and sustain it? What factors influence marriage immigrant women’s resilience, and how do they do so? Immigrant participants were asked about the difficulties they faced in South Korea as marriage immigrant women, factors that improve life in the country, and their experience of mental health promotion programs there to explore the context of their lives and their processes of resilience. Program manager informants were asked about the difficulties marriage immigrant women face in South Korea, factors that improve or harm their life in the country, and the manager’s experience of mental health promotion programs with immigrant women for triangulation. Field notes were made throughout the interviews. The interview questions were developed in discussion with qualitative research experts and based on results from literature reviews of resilience theory and mental health program evaluations [20]. The interview questions were pilot tested.

Furthermore, sociodemographic information of participants was gathered in order to characterize them. Interviews lasted 60 ~ 90 min, were recorded in mp3, and were transcribed by the author within 2 days. With regards to credibility, recording. and transcribing the interviews helped ensure the quality of the data [32]. To enhance the transparency and transferability, all stages of data collection and analysis were described. The marriage immigrant participants were able to communicate sufficiently in the Korean language to be understood. Interview participants had the opportunity to request an interpreter, but none did.

### Data analysis

Resilience is the result of individuals being able to interact with their environments and the processes that either promote well-being or protect them against the overwhelming influence of risk factors [33]. Difficulties are a prerequisite for resilience, and positive adaptation, recovery, and growth are the outcome indicators. Modern definitions at the individual, family, neighborhood, and community levels can extend interest from factors that support or hinder the resilience process at the individual level to the critical role of the environment surrounding the individual, including services [34]. According to this theoretical framework, the resilience of immigrant women can be explained by various factors such as social policy, neighborhood and social context, family, and personal development level.

This framework provides an analytic lens that enables the researcher to critically examine these informants’ narratives and identify the factors that influence marriage immigrant women’s resilience and its process when facing difficulties focusing on assets, strength, and supportive aspects of individuals’ environment. With a resilience theory framework as a lens, the author read and reread the transcripts to gain familiarity with the data. An initial reading of data identified critical text fragments and meaning before a thematic content analysis was conducted. Four thousand, seven hundred seventy-nine codes were initially established on the objective of the study. This allowed the generation of mixed categories, a theme analysis to find core categories, a comparison of categories and concepts, and the identification of similarities and differences between cases. Thereafter, open coding and axial coding were conducted. Based on the 4779 codes, similar statements were grouped and were finally compressed into 236 codes. By comparing and contrasting the codes across the cases, the author developed them into higher-order categories and presented them to other qualitative research experts. Analysis was conducted through discussions and continuously referring to the data. Through the categorization process, which compared and contrasted the relevance of the generated codes, the author derived 21 subcategories, which were further compressed into 13 categories. In these 13 categories, five common themes were derived by structuring common attributes.

### Research ethics

This study received the final approval (IRB No. 1503 / 002–004) of the Institutional Committee of Research Ethics of Seoul National University in February 2015. All study procedures involving human participants were in accordance with the ethical standards of the institutional committee and the 1964 Helsinki Declaration and its later amendments or comparable ethical standards. The researcher did not attempt any intervention concerning the experience of the research participants. There was no pressure by the researcher nor the program manager for the immigrant women to participate in this study. Written information about the goals of the study, the interview procedure, informant’s rights, and ethical considerations were issued to the women. Participation was voluntary and written informed consent was given by every participant, which guaranteed the informants’ rights, confidentiality, and anonymity. This manuscript was prepared in accordance with the COREQ standards for qualitative research reports.

## Results

Twenty-three participants, including 12 marriage immigrant women and 11 program managers, were interviewed. Table 1 describes the background characteristics of the participants who were interviewed. Twelve immigrant women were aged between 25 and 47 years (mean 35.8 years) and had lived in South Korea between 5 and 19 years (mean 12.5 years). Five immigrant women had just one child, while the other seven had two or three children. Eleven program managers were aged between 30 and 53 years (mean 39.6 years) with professional specialties in counseling or art therapy. They had been working with immigrant women between 2 and 25 years (mean 10.3 years).

**Table 1:** The sociodemographic characteristics of participants (*n* = 23)

Characteristics		Immigrant woman *n* = 12		Program manager *n* = 11			
				Female		Male	
		No.	%	No.	%	No.	%
Age	20–29	2	16.7	–	–	–	–
	30–39	8	66.6	6	54.5	1	9.1
	40–49	2	16.7	2	18.2	–	–
	50 and over	–	–	2	18.2	–	–
Marriage	Yes	12	100	8	72.7	0	–
Employment	Yes	5	41.7	10	90.9	1	9.1
Education	Under elementary	2	16.7	–	–	–	–
	Middle high or High	6	50.0	–	–	–	–
	Over College	4	33.3	10	90.9	1	9.1
Origin Country	Vietnam	3	25.0	–	–	–	–
	China	5	41.7	–	–	–	–
	Mongolia	1	8.3	–	–	–	–
	Japan	3	25.0	–	–	–	–
	Korea	–	–	10	90.9	1	9.1
Years of living in the host country	Less than 5 years	1	8.3	–	–	–	–
	6–10 years	3	25.0	–	–	–	–
	11–15 years	5	41.7	–	–	–	–
	More than 16 years	3	25.0	–	–	–	–
Years of working with migrants	Less than 5 years	–	–	3	27.3	1	9.1
	6–10 years	–	–	3	27.3	–	–
	11–15 years	–	–	2	18.2	–	–
	More than 16 years	–	–	2	18.2	–	–
Number of children	1	5	41.7	–	–	–	–
	2	5	41.7	–	–	–	–
	3	2	16.6	–	–	–	–
Type of institution	Public funded	12	100	10	90.9	1	9.1
Total		12	100	10	90.9	1	9.1

**Fig. 1:**
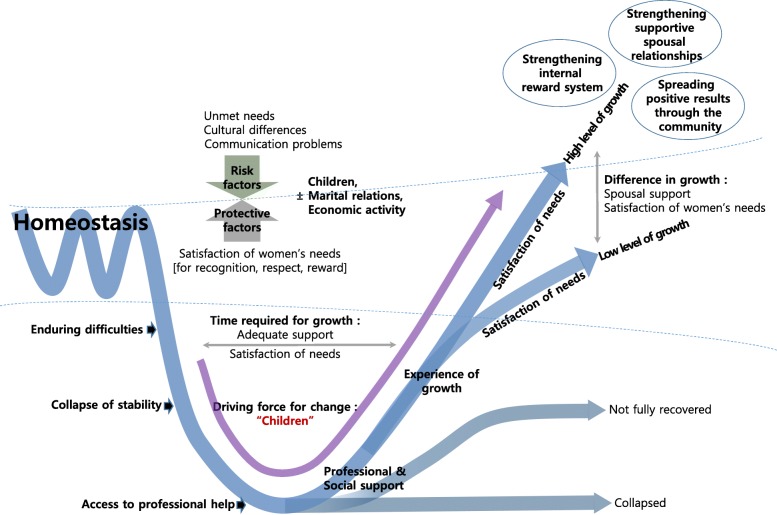
The resilience model of marriage immigrant women in mental health promotion services

From the analysis of the interviews, five themes emerged to represent marriage immigrant women’s process of resilience, its related factors, and their growth and ability to thrive (Fig. 1). This is described in more detail in Table 2. The discourse extracts in the text are labeled to indicate the source of the data (MIG: Marriage immigrant woman, number, and the number of years in Korea; PRO: Program manager, number, and the number of years spent by the information provided in their profession).

**Table 2:** Themes and categories of marriage immigrant women’s resilience process

Themes	Categories	Sub-categories
1.Staged process of growth	Enduring difficulties	Difficulties due to cultural differences
		Communication difficulty
		Conflicts due to family relationships
		Financial problems
	Collapse of stability	Collapse of stability within 1 ~ 2 years
		Mental illness
	Access to professional help	Informative support from friends
		Deciding whether to get help
	Professional and social support	Meeting the needs of recognition, respect, and reward
		Developing skills in expression and communication
	Experience of growth	Experience of positive emotion
		Change of view
		Efforts to strengthen growth
2.Absence of partner support	Close yet far-away husbands	
	Support of a friend during collapse	
3.Children as the driving force for change	Positive effects of children	
	Negative impacts of children	
4.The need for economic activity	Benefits from economic activity	
	Loss from economic activity	
5.Factors affecting difference in growth	Spousal support	
	Satisfaction of women’s needs for recognition, respect, and reward	

### Theme 1. Staged process of growth

Five categories of ‘enduring difficulties,’ ‘collapse of stability,’ ‘access to professional help,’ ‘professional and social support,’ ‘experience of growth’ are the order of the resilience process under ‘Staged process of growth’ theme.

#### Enduring difficulties

Differences in culture and difficulties in communication were found to be key factors influencing informants’ mental health. Cultural differences mostly included eating habits, ways of managing living spaces, parenting styles, and clothing, which led to family conflicts.

*“Mom in-laws say men are king and sky. Women are as low as the land. It’s so unjust and strange” (Marriage immigrant woman [Mig3, 11y]).*

*“My husband does not prepare my baby’s meal. He doesn’t even feed her. Moreover, he said there is nothing wrong with him. He thinks he is a good father.” (Mig10, 9y)*.

*“It is so hard. We (my husband and I) fight almost three times a month. I am so sick of him screaming and yelling at the children” (Mig3, 11y)*.

Participants reported that neither the marriage immigrant women nor their Korean family members felt their own culture was respected. Since their Korean language level was low, this often led to communication problems and confusion.

*“I could only understand one word, so I had to guess. It’s hard to guess. Conflicts kept happening” (Mig8, 8y).*

The participants, including both immigrant women and program managers, indicated that many immigrant wives are forced to speak only in Korean and to follow Korean culture, and this made them feel neglected and discriminated against.

*“Korean family members say these women are from savage and poor countries. They look down on them” (Program manager [Pro8, 4y]).*

As migrants without full citizenship, until they have acquired the necessary language and cultural skills, as well as legal status in their own right, these women have to rely on their husbands to make applications for change in status, fill in forms, or otherwise deal with authority. Lack of a common language restricts communication between husband and wife and increases the isolation of immigrant wives. Husbands may not want their wives to meet and socialize with their ethnic community members, as they are concerned to ensure that the wife learns to become an acceptable wife for the Korean family. Korean men’s unrealistic expectations of their immigrant wives’ behavior often cause conflict in marriage and lead to abusive domestic violence.

*“I was not allowed to go out for the first one to two years in Korea. My husband and mom-in-law were worried that I would run away if I go out” (Mig3, 11y).*

*“There were husbands saying that they bought a bride for child, child bearing, descendants … and these men treat the wives bad and often neglect them” (Pro8, 4y).*

Economic difficulties were also noted as a major negative factor in the mental health of the marriage immigrant women. Most of the spouses mentioned had a low income, and many were unemployed. As a result, immigrant women could not support their home country’s family financially and suffered from an economic burden with their Korean family.

*“My parents think, ‘My daughter is internationally married. She will pay me.’ So, I have to send money. It is always difficult” (Mig8, 8y).*

#### Collapse of stability

Most immigrant participants reported mental health problems, including emotional difficulties, stress, anxiety, and depression. Several immigrant informants said they had struggled with suicidal ideation during their first or second year in South Korea.

*“I was stressed out, and I wanted to die. I really did not want to live anymore” (Mig10, 9y).*

Migration reduces the capacity of individuals to act independently and increases the vulnerability of women until they have legitimate status in their own right as well as cultural and language skills. Immigrant women commonly addressed that the first one to 2 years was the hardest time to adjust in the new country.

*“The first two years in Korea was the toughest time in my life. I tried so hard to learn the Korean language to communicate with my husband, but he did not trust me and locked me at home” (Mig8, 8y).*

*“It was so hard after arriving in Korea. It was hard enough that I could die. I was so depressed and could not see any future ahead of me. For about one year, it was so tough to adjust” (Mig10, 9y).*

Program manager informants also addressed that immigrant women struggle hard, especially in the first one to 2 years in South Korea.

*“The first two years are the hardest time. They have to learn Korean and adjust to a new life. Complaints build up and suddenly explode. Then they run away or want a divorce” (Pro2, 15y).*

Though their initial threshold for problems might be high, due to lower levels of social support, family conflict, traumatic events like domestic violence, and greater social isolation, stress had built up until the marriage immigrant women’s mental stability collapsed. Several immigrant women explained that their self-esteem had gotten progressively lower, and they could not manage their sudden and extreme tempers.

*“I felt incompetent all day long. There was nothing I could do, so I got sick and upset. My complaints festered and exploded” (Mig1, 5y).*

#### Access to professional help

Access to professional services in a new country was challenging for immigrants. The marriage immigrant women came from countries with no public counseling system and were used to experiencing stigma towards mental health problems. Thus, they did not know what to do or where to get help when suffering from severe mental health problems. Most participants who had experienced a mental health problem utilized informative support from other immigrant friends, not from their Korean family.

*“I have a married immigrant friend, and she has a lot of information. She told me about the program” (Mig4, 15y).*

These friends could explain the benefits of professional intervention and help them navigate the services. One immigrant woman explained that she could get professional assistance from police while arranging services for a domestic violence victim:

*“I didn’t know that there is a shelter for women. He kept hitting me, and I didn’t have a place to hide from him. I called the police for the first time. Then the counselor came along with the police” (Mig3, 11y)*.

The marriage immigrant women preferred in-center programs, while they stated their Korean family preferred home-visit programs, ostensibly to prevent them from “running away.” When a professional service was available, marriage immigrant women had the option of getting help if necessary.

*“At first, I was very angry, and I said ‘I’m going back to Vietnam, I’m going to divorce.’ But when I was pregnant, I began to think about how I should survive. So I thought I should get help and learn to live” (Mig8, 8y).*

However, not all women made use of these services. Women with children were found to be more likely to use the services than women without children.

#### Professional and social support

Marriage immigrant women felt frustrated when they were not recognized, rewarded, or respected by their Korean families. Social and emotional support from professional services met those needs restored emotional stability and self-esteem.

*“[The program] has changed my life. It was helpful that someone listened to me and treated me with respect. I felt relaxed” (Mig3, 11y).*

Professional help included counseling, couple counseling, music therapy, art therapy, self-help group activities, and community service activities. Counselors and therapists gave advice, therapy, useful information, full attention, listened to their stories, and empathized with them. Through self-help group activities and volunteer activities, new support systems were formed. Marriage immigrant women also learned healthy communication skills to express their thoughts and feelings appropriately.

*“I used to think of myself as alone and didn’t talk. Now I say what I think, and my husband says, “Thank you very much for your words.” [Things are] very good with my husband now” (Mig11, 5y).*

#### Experience of growth

Participants explained that professional help reinforced marriage immigrant women’s self-efficacy:

*“Really passive women became leaders full of confidence. I was really surprised” (Pro10, 4y).*

and helped them resolve family conflicts through healthy communication. In addition, as they understood and sympathized more with the differences of their counterparts, they were better able to understand the position of the Korean family:

*“I understood my husband while counseling. At first, I only voiced my opinions. However, I realized how frustrating it was for my husband. Now that we understand each other, things have changed a lot” (Mig8, 8y).*

Rapport with professional staff and self-help group members created secure support networks and gave the immigrant women strength to work through difficulties. They saw themselves as “grown from the past.” Furthermore, as they experienced growth through the programs:

*“I never knew what I was capable of, and now I think I’m starting to fly” (Mig4, 15y).*

, they made efforts to strengthen their internal reward systems continuously. In particular, they enjoyed the praise and recognition that come from volunteer activities.

### Theme 2. Absence of partner support

Few of the immigrant women in this study got the help they needed from their spouses.

*“My husband is the closest person to me, but only physically. We have many conflicts. I can’t communicate with him. It is so hard” (Mig8, 8y).*

As they were financially dependent on their husbands, this left them with less control over their lives and mental health.

*“The economic power is almost always with their husband or their parents-in-law. Because these immigrant women don’t have pocket money, they can’t live their own life at all. That is why they struggle to get a part-time job” (Pro50, 8y).*

Thus, friends were important to participants in two ways. Firstly, they were able to recognize collapses in stability in these married women. Secondly, they informed professional services and helped the participant access these services if necessary.

*“I came to the center with my friend. I did not know about the center or how to get there” (Mig8, 8y).*

These friends included neighbors and people from Social Networking Service (SNS) like Facebook, parent meetings, or religious centers.

### Theme 3. Children as the driving force for change

All participants mentioned the importance of childcare, and a decision to get help was often made as a result of childcare responsibilities.

*“After I gave birth, I believed I needed to change for my child. I had to try” (Mig6, 15y).*

After getting pregnant or giving birth, the married women reconstructed their foci to their child and changed their attitudes and strategies towards difficulties from passive to active.

*“It was so hard that I wanted to die. I thought a lot about killing myself, but I became a mother. I had to raise my child somehow! I had to be strong” (Mig8, 8y).*

In addition, a child gave them a sense of security and stability as it guaranteed they would be able to stay in South Korea indefinitely. Children also gave psychological comfort to the married women*.*

*“She [My child] takes care of me. I said to her, ‘it hurts,’ and she gently rubs her hand against my forehead. I felt relaxed” (Mig11, 5y).*

The results showed that children could also cause the marriage immigrant women problems. These results were commonly addressed by program manager informants. In cases of disagreement among Korean family members regarding childcare, child maladaptation, and child discrimination, the women also suffer. Several program manager informants in the study mentioned that the child neglected and discriminated against immigrant women as the Koreans do.

*“The children ignore their mother like their grandma does. They learn this without knowing it, and they don’t respect their mother” (Pro8, 4y).*

### Theme 4. The need for economic activity

Economic activities, which are known to be protective factors in resilience theory [35], appeared to affect mental health in both positive and negative ways, related to the context in which married women had to work for a living.

*“There are few people who can afford to live. And there are a lot of people who are in debt, so they have to work” (Pro1, 25y).*

The marriage immigrant women felt a sense of duty to support families in their home country. One participant stated that.

*“The reason why immigrant women are forced to do economic activities is … to send money to their families in their home countries” (Pro6, 5y)*.

However, they were financially insecure in South Korea and had to work to earn money to support their families in South Korea as well. There are also benefits from this economic activity, such as supporting one’s family financially, developing one’s career, and earning the respect of one’s Korean family:

*“My life becomes energetic, and I find work that strengthens my talent, and my children get to respect me” (Mig7, 13y)*;

however, this economic activity has a cost. The balance of work and household duties made them feel doubly burdened.

*“I was stressed a lot. Not stressed at work, but afterward. It was too hard for housekeeping” (Mig8, 8y).*

However, some women in the study were satisfied with their role in society and their financial independence within the family.

### Theme 5. Factors affecting difference in growth

The level of growth or and ability to thrive varied among participants and appeared to be influenced by satisfaction levels of women’s needs for recognition, respect, and reward and support levels of the partner. By recognizing their value through various social activities, marriage immigrant women were able to express their feelings and thoughts with healthy communication and restore their self-esteem and confidence. The support of the husband made a difference in the degree of growth. Adequate support also sped up the time required for growth. If a spouse understood and supported the adaptation efforts of the immigrant women, positive marital relations were strengthened, and a high-level growth was achieved in a short time.

*“I wanted him to know my heart and not ignore it. If my husband gives me his heart, I can bear it. That the most important thing” (Mig3, 11y).*

The results also revealed that individuals’ changes had a positive effect on family relationships and the community.

*“I have a desire to go out and help others through volunteer activities. It is worthwhile, and I feel good. I like to receive gratitude and praise” (Mig6, 15y).*

## Discussion

In this study, the author aimed to identify the factors that influence marriage immigrant women’s resilience and its process when facing difficulties. In doing so, factors relating to seeking professional help were also identified. The findings indicated that the order of the resilience process follows: enduring difficulties, collapse of stability, access to professional help, professional and social support, and experience of growth. A combination of the staged process of growth, absence of partner support, children as a driving force for change, the need for economic activity, factors affecting difference in growth all affect marriage immigrant women’s resilience. Common difficulties that these women face are cultural differences and communication problems due to the lack of language proficiency. A previous study also reported that one of the main difficulties faced by immigrant women is language and communication [36]. It should be noted that often, marriage immigrant women are passive in seeking help for mental health problems, as described in a previous study [37]. As support from the home country family weakened while living in Korea, their vulnerability to mental health problems heightened. Thus, accessibility to professional help becomes extremely important in this context.

In general, immigrants are evaluated as vulnerable due to a lack of access to health information compared to indigenous peoples [38]. Marriage immigrant women experience structural barriers when accessing professional services as a result of their position as immigrants in society [39]. The women lack information about public services and are often reliant on other immigrant women for information. These circumstances delay intervention and cause more complex mental health problems, forcing marriage immigrant women into a subordinate position with less control over their own lives. Migrants are often also unfamiliar with the overall system and language of the immigration country, so experience significant difficulty obtaining necessary health information [40]. It is imperative that access to professional services is improved to empower immigrant women. The main task of marriage immigrants is to overcome adversities in managing cross-cultural marriage and life in Korea in the first few post-migration years [19]. According to this study’s findings, this professional help should be available within 2 years of migration. This supports research from other countries that suggests intervention should not be delayed over 3 years [41]. How and when marriage immigrant women receive service information, and professional help is essential since mental health problems can be easily resolved at an early stage [6, 42] As mentioned in Chang and Wallace’s work [19], effective integration programs in the first few post-migration years may identify at-risk transnational couples, and improve marriage migrants’ health. Translation and interpretation are significant parts to play in facilitating access to support and services, and accurate, high-quality services of translation and interpretation should be a crucial step in promoting services to the women [4]. By increasing awareness of available services, immigrants can achieve positive changes in their lives [43]. The use of lay health providers or assistants is essential to prepare this population for the empowerment process [44].

Meeting marriage immigrant women’s needs for recognition, respect, and reward, in the form of happiness, well-being, encouragement, and financial independence, and strengthening supportive spousal relationships are the key intervention strategies for improved resilience. Many of the participants in this study find a supportive relationship with counselors and therapists where they can discuss their concerns, thoughts, and feelings and are encouraged to learn communication skills. The national hotline and counseling services carrying out in the client’s native language to ensure that the woman feels able to express herself as she wishes, are recommended [4]. However, although this type of intervention is sufficient, the role of the partner is a lot bigger in resilience. The degree of respect, acceptance, and attention received from others who are considered to be meaningful has the most significant impact on the development of self-esteem [45]. Since their Korean spouses are usually the most important and meaningful people to marriage immigrant women, being recognized, respected, and rewarded by the spouse and building a strong supportive relationship with them decreases the resilience process time and leads the women to a high level of growth. If women feel marginalized and stigmatized, supporting their integration and protecting them from risk factors of mental health will be very challenging, so professionals may need to work with the Korean families as well as with the wives themselves [4]. The utilizing bilingual healthcare providers or assistants having same country of origin with the women in the multicultural centers would be useful to provide culturally sensitive and intimate care, patient education, and friendship for immigrant women [46]. As such, partner intervention regarding fostering a positive marital relationship and its fees should be addressed at the system level. Moreover, family educational programs should be provided to promote egalitarian spousal relationships and prevent domestic violence [45].

Since 2006, over 200 multicultural centers have been established countrywide in Korea to provide services to marriage immigrant women [47]. These centers have become significant places for immigrant women to meet and make friends, access to services for integration, find a source of support for the women and their families, start occupational training and language learning, and cross-cultural communication [4]. A previous study addressed the importance of social health buffering marital distress and acculturative stress of immigrants by enhancing cohesion and decreasing family conflict [48]. Hence supporting the self-help groups of immigrant wives can have a significant role to break the isolation of these women in the local communities by programs or services working to give women some support and security [49–52]. In addition to obtaining social support, regular social engagement outside the family may also prevent immigrant women from integration-related maltreatment from husbands or in-laws [19]. A recent survey presented that more than half of marriage immigrant women’s social participation appeared only within co-ethnic contacts, with a weak connection with Koreans [53]. Given the decisive role of cross-group relationships with majorities in minority’s well-being [54], community efforts to increase opportunities for marriage immigrant women to interact with Koreans will benefit their resilience. Government institutions can have a considerable effect on how vulnerable immigrant women are as wives in a different country, and the long-term goal of agencies should be to change legislation to provide women more secure status in a new country from the moment they become part of a permanent partnership with a citizen [4].

This is the first study, to the author’s knowledge, that found that the children can be the driving force in tackling the difficulties of marriage immigrant women. Psychological resources, such as child-care responsibility and the tight bond with a child, play a role as catalysts for improvements to immigrants’ mental health and buffers to mediate stressors [55]. These results confirm that the needs for a relationship plays a vital role in promoting intrinsic motivation [56]. Personality traits and psychological resources have been the main emphasis in existing resilience research [57–61]; however, the results of this study show that the protective factors of resources at the family and community level are more influential on resilience than individual resources concerning the mental health of marriage immigrant women. Another noteworthy finding is that economic activity plays positive and negative roles in the mental health of these immigrant women as it is both a means of solving financial difficulties and a stressor that makes it challenging to balance work and family life. The resilience factor, therefore, needs to be interpreted in a way that reflects this cultural and social context [62], and economic activities should be supported structurally for the independence and autonomy of these women.

Opinions of scholars vary on whether to view positive results after difficulty as a recovery to the previous state or an improved state [63]. The discoveries of this study suggest that for marriage immigrant women, resilience results should be regarded as growth beyond recovery to the stable state, in that they overcame difficulties and then achieved further internal growth through various protective factors, such as strengthening their support system. In this process, their attitudes, thoughts, coping styles, and communication styles changed positively. These positive changes correspond to areas of growth that include appreciation of life, intimacy of relationships, and personal strengths [31, 64]. To sustain and strengthen growth in resilience in marriage immigrant women, interventions should be considered for individuals, which include woman, her spouse, and family members, to undertake meaningful activities in a supportive and collaborative environment in the community [65]. In turn, the positive results of resilience may then spread through the community.

One limitation of this qualitative research could be that marriage immigrant women who participated in the in-depth interviews may have more resources at their disposal than other marriage immigrant women in that they had already experienced positive resilience results. However, as the purpose of this study was to analyze marriage immigrant women who experienced positive results of resilience, and to identify resilience factors, processes, and its context, it is not necessary to consider the possibility of selective bias against the study results. Further studies should address the resilience of the mental health of marriage immigrant women who have few protective factors in the early stages of migration. Another limitation of this research is that the fieldwork was conducted three to 4 years ago; there has now been a policy change in South Korea that basic Korean proficiency is required for marriage immigration. Since this policy change may affect immigrant women’s resilience process, further study should be addressed to identify the effect of language proficiency in resilience. Further research should explore how gender, women’s social position, power, cultural beliefs and values, social and institutional ideologies intersect to influence marriage immigrant women’s resilience, mental health, and quality of life. The author from South Korea having different cultural backgrounds to immigrant women and having the role of a researcher may have been considered an outsider to the immigrant women. Nevertheless, with their trust in program managers who introduced the author to them and their willingness to participate in delivering their voice to change the society/policy, the informants may have been comfortable in discussing topics related to their lived experience in South Korea. Findings are dependent on the subjective interpretations of the researcher. Although the author had responsibility for data collection and analysis, the data was discussed with the other qualitative research experts in migration and health study to enable to explore different perspectives and contribute to the credibility of the findings [66].

## Conclusions

This study aimed to show how marriage immigrant women become resilient when facing a number of difficulties and what the key influential factors to resilience are. The contextual factors that influence immigrant women’s coping strategies for mental health problems were highlighted. Several key factors that play positive and negative roles in resilience were identified at individual, social, cultural, and structural levels. While these related factors are in line with previous research with other immigrant women, this detailed analysis adds insights into how these factors influence its growth process. Before stability collapses, it is important for marriage immigrant women to access professional services within 2 years of migration. Building a supportive relationship with their Korean spouses and meeting their needs are key strategies for intervention to these women, while information barriers should be addressed at a structural level in order to improve the mental health of these women.

## Data Availability

The datasets generated during the current study are not publicly available due to the sensitive and personal nature of the information contained in the data. Data may be available from the current authors, with restrictions and following ethical approval.

## Abbreviations

**MIG**: **Marriage immigrant woman**
**PRO**: **Program manager**
**SNS**: **Social Networking Service**
